# Patient's Origin and Lifestyle Associated with CTX-M-Producing *Escherichia coli*: A Case-Control-Control Study

**DOI:** 10.1371/journal.pone.0030498

**Published:** 2012-01-27

**Authors:** Marie-Hélène Nicolas-Chanoine, Vincent Jarlier, Jérôme Robert, Guillaume Arlet, Laurence Drieux, Véronique Leflon-Guibout, Cédric Laouénan, Béatrice Larroque, Valérie Caro, France Mentré, the study Group Coli β

**Affiliations:** 1 Service de Microbiologie, Hôpital Beaujon AP-HP, Clichy, France; 2 Faculté de Médecine D. Diderot, Paris, France; 3 Institut National de la Santé et de la Recherche Médicale, U773, Centre de Recherche Biomédicale Bichat-Beaujon (CRB3), Université Paris, Paris, France; 4 Laboratoire de Bactériologie Hygiène Hospitalière, Hôpital Pitié Salpêtrière AP-HP, Paris, France; 5 EA 1541, Université Pierre et Marie Curie-Paris, Paris, France; 6 Service de Bactériologie, Hôpital Tenon AP-HP, Paris, France; 7 Equipe Opérationnelle en Hygiène Hospitalière, Hôpital Charles-Foix AP-HP, Ivry-sur-Seine, France; 8 UMR-S 738 INSERM, Université D. Diderot, Paris, France; 9 UF de Biostatistiques, Hôpital Bichat AP-HP, Paris, France; 10 Unité d'épidémiologie et de Recherche Clinique, Antenne URC Paris Nord, Hôpital Beaujon AP-HP, Clichy, France; 11 Plateforme Génotypage des Pathogènes et Santé Publique, Institut Pasteur, Paris, France; Public Health Agency of Barcelona, Spain

## Abstract

**Background:**

Global dissemination of *Escherichia coli* producing CTX-M extended-spectrum β-lactamases (ESBL) is a public health concern. The aim of the study was to determine factors associated with CTX-M- producing *E. coli* infections among patients hospitalised in the Assistance Publique-Hôpitaux de Paris, the largest hospital system in France (23 000 beds), through a prospective case-control-control study.

**Methods/Principal Findings:**

From November 2008 to June 2009, 152 inpatients with a clinical sample positive for CTX-M-producing *E. coli* (cases), 152 inpatients with a clinical sample positive for non ESBL-producing *E. coli* on the day or within the three days following case detection (controls C1), and 152 inpatients with culture-negative clinical samples since the beginning of hospitalisation and until three days after case detection (controls C2) were included in ten hospitals of the Paris area. Factors studied were related to patient's origin, lifestyle and medical history as well as care during hospitalisation. Those independently associated with CTX-M-producing *E. coli* were determined. Three independent factors were common to the two case-control comparisons: birth outside of Europe (cases *vs* C1: OR_1_ = 2.4; 95%CI = [1.3–4.5] and cases *vs* C2: OR_2_ = 3.1; 95%CI = [1.4–7.0]), chronic infections (OR_1_ = 2.9; 95%CI = [1.3–6.9] and OR_2_ = 8.7; 95%CI = [2.0–39.7]), and antibiotic treatment between hospital admission and inclusion (OR_1_ = 2.0; 95%CI = [1.0–3.8] and OR_2_ = 3.3; 95%CI = [1.5–7.2]). Cases were also more likely to be (i) functionally dependent before hospitalisation than C2 (OR_2_ = 7.0; 95%CI = [2.1–23.5]) and (ii) living in collective housing before hospitalisation than C2 (OR_2_ = 15.2; 95%CI = [1.8–130.7]) when CTX-M-producing *E. coli* was present at admission.

**Conclusion:**

For the first time, patient's origin and lifestyle were demonstrated to be independently associated with isolation of CTX-M-producing *E. coli*, in addition to health care-related factors.

## Introduction

*Escherichia coli* is a universal commensal of humans and several animal species. It is also one of the most common *Enterobacteriaceae* causing extra-intestinal infections [1]. Because of these ecological features, *E. coli* is constantly exposed to antibiotics and developing mechanisms of resistance to antibiotics. Since 2000, *E. coli* isolates resistant to extended-spectrum cephalosporins by production of extended-spectrum β-lactamases (ESBL) have emerged worldwide in both community and hospital settings [2]. Unfortunately, ESBL-positive isolates are also commonly resistant to fluoroquinolones and cotrimoxazole, two antibiotics widely used to treat community-onset urinary tract infections (UTI) [2]. The dissemination of these multidrug resistant (MDR) *E. coli* isolates occurred concomitantly with the emergence of a new ESBL family called CTX-M and derived from the chromosomal β-lactamases of *Kluyvera* spp., an environmental *Enterobacteriaceae* [3]. Among the numerous plasmid-mediated CTX-M enzymes described to date, CTX-M-1, CTX-M-14 and CTX-M-15 currently predominate [2], [3]. Moreover, a widely disseminated lineage of virulent *E. coli*, designated sequence type ST131 according to multilocus sequence typing and producing CTX-M-15 has been identified [4], [5]. The epidemiology of CTX-M-producing *E. coli* is complex because these isolates have become ubiquitous: in the community and in the hospital in many countries [5], in animals [6], [7], and also in the environment [8]. Therefore, identifying patients at risk for harbouring ESBL-producing *E. coli* among all infected patients, especially those with community-onset infections, is of paramount importance for both choice of treatment and limitation of the diffusion of these resistant strains.

The aim of the study was to determine factors associated with CTX-M- producing *E. coli* infections among patients hospitalised in the Assistance Publique-Hôpitaux de Paris (AP-HP), the largest hospital system in France (23 000 beds) located in Paris area, through a prospective case-control-control study.

## Results

### Participants

**Figure 1:**
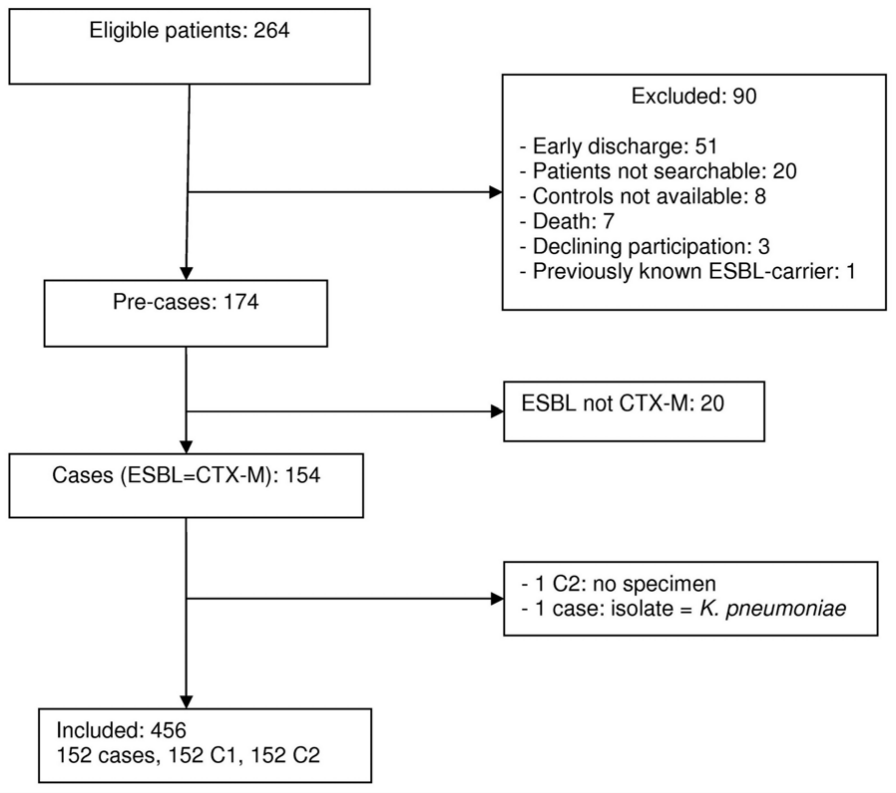
Flow chart for inclusion.

Among the 264 eligible patients (patients with a clinical isolate of *E. coli* producing an ESBL), 174 were pre-included and 90 excluded as indicated in Figure 1. Among the 174 pre-cases, 20 (11.5%) with *E. coli* producing an ESBL other than CTX-M were excluded. Therefore, 154 cases had to be included in the study. However, two cases were secondly removed because one had a corresponding control C2 with specimen sampled outside the required time frame, and for the other one, the isolate producing CTX-M was *Klebsiella pneumoniae*. Finally, 152 cases with two controls for each case (304) were included in the study.

A majority of cases and controls were hospitalised in short-term facilities (cases: n = 136, C1: n = 139, C2: n = 139) and essentially in adult medical wards (cases: n = 96, C1: n = 99, C2: n = 108). Hospital death occurred in 12% of cases, 7% of C1, and 5% of C2 patients. The difference was statistically significant between cases and C2 (p = 0.02). Overall, 62% of the samples were from the urinary tract, 26% from various deep sites (blood, surgically-sampled specimens, respiratory and ascetic fluid) and 12% from other sites. There was no statistical difference among the type of clinical sample according to cases and controls.

### CTX-M enzymes

CTX-M-15 accounted for 51% of the CTX-M enzymes followed by CTX-M-1 (24%), CTX-M-14 (12%), and CTX-M- 27 (4%). The remaining CTX-M consisted of six other CTX-M enzymes.

### First case-control study

When cases were compared to C1, cases were more likely than C1 to be born in a country outside of Europe (odds ratio [OR] = 2.3; 95% confidence interval [CI] = 1.3–4.0) (Table 1). Among the 52 cases born in a country outside of Europe, 34.5% were born in Asia, 34.5% in North Africa, 27% in sub-Saharan Africa and 4% in South America. In addition, cases were more likely than C1 to live outside of Europe (OR = 5.0; 95%CI = 1.1–22.8) (Table 1); to have been hospitalised ≥10 days (OR = 3.0; 95%CI = 1.6–5.6) within the last six months; to have recurrent UTI or chronic skin infections (OR = 3.4; 95%CI = 1.6–7.2), and invasive devices within the last six months (OR = 1.9; 95%CI = 1.1–3.1); to have received an antibiotic treatment within the last month (OR = 2.6; 95%CI = 1.5–4.6), especially ≥5 days (OR = 2.2; 95%CI = 1.1–4.4), cotrimoxazole (OR = 10.0; 95%CI = 1.3–78.8) or extended-spectrum cephalosporins (OR = 5.5; 95%CI = 1.2–24.8) (Table 2). Regarding the current hospitalisation (Table 3), cases were more likely than C1 to have had intravascular devices (OR = 2.0; 95%CI = 1.1–3.6) and received antibiotics between admission and inclusion (OR = 2.6; 95%CI = 1.4–4.6), especially for a duration ≥5 days (OR = 3.3; 95%CI = 1.6–6.7). On the opposite, cases were less likely than C1 to eat raw meat (OR = 0.5; 95%CI = 0.3–0.9), and to have contact with pets or livestock (OR = 0.5; 95%CI = 0.3–0.9) (Table 2). Of note, there was no statistical difference between cases and C1 regarding the characteristics of household members, *i.e.* number, age, occupation in healthcare facilities, and medical history.

**Table 1:** Univariate analysis of demographic and lifestyle factors associated with a CTX-M-producing *E. coli* clinical isolate through a case (patient with a CTX-M producing *E. coli* isolate) - control (C1: patient with a non-ESBL-producing *E. coli* isolate) - control (C2: patient with negative clinical samples) study.

Factor	Case No. (%)	C1 No. (%)	Odds ratio (95% CI)	P value	C2 No. (%)	Odds ratio (95% CI)	P value
*Demographic data*							
Age (mean ± SD) in years	64±25	64±22	1.0 (1.0–1.0)	0.7	61±22	1.0 (1.0–1.0)	0.1
Age <15 years	9 (6)	3 (2)	7.0 (0.9–56.9)	0.07	3 (2)	7.0 (0.9–56.9)	0.07
Age ≥65 years	84 (55)	85 (56)	1.0 (0.6–1.6)	0.9	77 (51)	1.3 (0.8–2.2)	0.3
Age ≥80 years	49 (32)	45 (30)	1.2 (0.7–2.1)	0.6	35 (23)	2.1 (1.1–4.0)	0.03
Female	99 (65)	93 (61)	1.2 (0.7–1.9)	0.5	79 (52)	1.7 (1.1–2.8)	0.02
Country of birth outside of Europe	51 (34)	27 (18)	2.3 (1.3–4.0)	0.003	29 (19)	2.0 (1.2–3.4)	0.007
Living in a country outside of Europe	11 (7)	3 (2)	5.0 (1.1–22.8)	0.04	6 (4)	2.0 (0.7–5.9)	0.2
*Lifestyle*							
Collective housing	27 (18)	21 (14)	1.8 (0.8–3.8)	0.2	10 (7)	10.4 (2.4–44.8)	0.002
Individual housing (>2 household members)	44 (29)	34 (22)	1.7 (0.9–3.0)	0.09	37 (24)	1.5 (0.9–2.6)	0.2
Live alone	34 (22)	40 (26)	0.8 (0.4–1.4)	0.4	56 (37)	0.4 (0.2–0.8)	0.005
Functionally dependent before hospitalisation.	49 (32)	36 (24)	1.7 (1.0–3.0)	0.07	20 (13)	5.1 (2.3–11.6)	<10^−4^
Patients not working	107 (84)	116 (92)	0.7 (0.4–1.2)	0.2	98 (91)	1.4 (0.8–2.5)	0.2
Retired patients	88 (69)	93 (74)	0.8 (0.5–1.4)	0.5	75 (69)	1.6 (0.9–2.6)	0.09
Consumption of - ≥7 raw vegetables/week	85 (69)	86 (68)	0.9 (0.4–1.6)	0.6	100 (77)	0.5 (0.3–1.0)	0.07
- poultry≥twice a week	71 (58)	71 (56)	1.1 (0.6–1.9)	0.8	79 (60)	0.9 (0.5–1.6)	0.7
- beef≥twice a week	78 (64)	87 (70)	0.8 (0.5–1.4)	0.5	92 (70)	0.7 (0.4–1.3)	0.3
Consumption of raw meat	37 (24)	57 (38)	0.5 (0.3–0.9)	0.02	57 (38)	0.5 (0.3–0.9)	0.01
Community meal	81 (53)	85 (56)	0.9 (0.5–1.5)	0.6	87 (57)	0.8 (0.5–1.4)	0.4
Practice of a sport	11 (7)	11 (7)	1.0 (0.4–2.4)	1.0	21 (14)	0.5 (0.2–1.0)	0.06
Pets or livestock	19 (13)	36 (24)	0.5 (0.3–0.9)	0.02	31 (20)	0.6 (0.3–1.1)	0.08
Travel abroad in the preceding 6 months (>14 days)	17 (11)	14 (9)	1.2 (0.6–2.6)	0.6	17 (11)	1.0 (0.5–2.0)	1.00

**Table 2:** Univariate analysis of medical history-related factors associated with a CTX-M-producing *E. coli* clinical isolate through a case (patient with a CTX-M producing *E. coli* isolate) - control (C1: patient with a non-ESBL-producing *E. coli* isolate) - control (C2: patient with negative clinical samples) study.

Factor	Case No. (%)	C1 No. (%)	Odds ratio (95% CI)	P value	C2 No. (%)	Odds ratio (95% CI)	P value
*Medical history*							
In the preceding 6 months							
- hospitalised	97 (63)	68 (45)	2.5 (1.5–4.1)	7×10^−4^	79 (52)	1.7 (1.1–2.8)	0.03
- hospitalised ≥10 days	61 (40)	35 (23)	3.0 (1.6–5.6)	4×10^−4^	43 (28)	1.9 (1.0–3.1)	0.02
- hospitalised <10 days	36 (24)	33 (22)	1.7 (0.9–3.5)	0.1	36 (24)	1.4 (0.6–2.9)	0.4
- hospitalised outside of France	8 (5)	2 (1)	4.0 (0.8–18.8)	0.08	1 (1)	8.0 (1.0–64.0)	0.05
- at least one invasive device	96 (63)	76 (50)	1.9 (1.1–3.1)	0.02	73 (48)	1.9 (1.2–3.0)	0.009
• urine drainage	47 (31)	28 (19)	2.0 (1.2–3.6)	0.01	14 (9)	4.8 (2.3–10.4)	<10^−4^
• mechanical ventilation	13 (9)	5 (3)	3.0 (1.0–9.3)	0.06	6 (4)	2.7 (0.9–8.6)	0.08
• intravascular devices	91 (61)	65 (43)	2.0 (1.3–3.3)	0.003	64 (43)	1.9 (1.2–3.1)	0.007
• colonoscopy, endoscopy,	37 (26)	29 (20)	1.5 (0.8–2.9)	0.2	28 (19)	1.5 (0.8–2.6)	0.2
Surgery during the last month	44 (29)	44 (29)	1.0 (0.6–1.7)	0.9	33 (22)	1.6 (0.9–3.0)	0.1
Prothesis within the last year	10 (6)	11 (7)	0.8 (0.3–2.1)	0.6	9 (6)	1.3 (0.5–3.8)	0.6
Antibiotic in the month preceding hospitalisation.	53 (35)	25 (16)	2.6 (1.5–4.6)	6×10^−4^	33 (22)	1.9 (1.1–3.1)	0.02
- cotrimoxazole	10 (7)	1 (1)	10.0 (1.3–78.1)	0.03	4 (3)	2.5 (0.8–8.0)	0.1
- fluoroquinolones	11 (7)	7 (5)	1.7 (0.6–4.6)	0.3	3 (2)	3.7 (1.0–13.1)	0.05
- extended spectrum cephalosporins	11 (7)	2 (1)	5.5 (1.2–24.8)	0.03	7 (5)	1.6 (0.6–4.1)	0.4
- penicillins	17 (11)	8 (5)	2.5 (1.0–6.4)	0.06	18 (12)	0.9 (0.5–1.9)	0.9
- ≥5 days	31 (20)	16 (10)	2.2 (1.1–4.4)	0.02	28 (18)	1.1 (0.6–1.9)	0.7
Nursing or physiotherapy before hospitalisation.	26 (17)	25 (16)	1.1 (0.6–2.0)	0.9	17 (11)	1.7 (0.9–3.4)	0.1
At least one co-morbidity	90 (59)	77 (51)	1.5 (0.9–2.4)	0.1	58 (38)	2.1 (1.4–3.4)	9×10^−4^
- ecurrent urinary tract or chronic skin infections	39 (26)	17 (11)	3.4 (1.6–7.2)	0.001	4 (3)	12.7 (3.9–410)	<×10^−4^
- obstructive bronchial pulmonary disease	7 (5)	4 (3)	1.8 (0.5–6.0)	0.4	4 (3)	1.7 (0.5–5.1)	0.4
- cancer	37 (24)	35 (23)	1.1 (0.3–1.8)	0.8	28 (18)	1.4 (0.8–2.4)	0.2
- diabetes	34 (22)	34 (22)	1.0 (0.6–1.8)	1.0	28 (19)	1.3 (0.7–2.3)	0.4

**Table 3:** Univariate analysis of current hospitalisation-related factors associated with a CTX-M-producing *E. coli* clinical isolate through a case (patient with a CTX-M producing *E. coli* isolate) - control (C1: patient with a non-ESBL-producing *E. coli* isolate) - control (C2: patient with negative clinical samples) study.

Factor	Case No. (%)	C1 No. (%)	Odds ratio (95% CI)	P value	C2 No. (%)	Odds ratio (95% CI)	P value
*Current hospitalisation*							
Transferred from another hospital	32 (21)	26 (17)	1.5 (0.7–2.9)	0.3	35 (23)	0.8 (0.4–1.7)	0.6
Mc Cabe score 2	34 (25)	20 (15)	1.8 (0.9–3.6)	0.1	22 (16)	1.6 (0.8–2.9)	0.2
Immunocompromised	51 (34)	42 (48)	1.3 (0.8–2.2)	0.3	43 (28)	1.3 (0.8–2.1)	0.3
Between admission and inclusion							
- ICU stay	31 (20)	19 (13)	1.8 (1.0–3.2)	0.06	16 (11)	2.9 (1.3–6.4)	0.01
- LTCF stay	35 (23)	38 (25)	0.5 (0.1–2.0)	0.3	29 (19)	3.0 (0.8–11.1)	0.1
- Invasive device during the last week	117 (77)	105 (69)	1.7 (0.9–0.3)	0.07	86 (57)	4.4 (2.2–9.2)	<10^−4^
• urine drainage	56 (37)	47 (31)	1.3 (0.8–2.1)	0.3	23 (15)	5.1 (2.4–10.9)	<10^−4^
• mechanical ventilation	24 (17)	17 (12)	1.5 (0.8–3.1)	0.2	7 (5)	9.5 (2.2–40.8)	0.003
• intravascular devices	112 (74)	95 (62)	2.0 (1.1–3.6)	0.02	82 (54)	4.0 (2.0–8.0)	<10^−4^
- Antibiotic receipt	81 (53)	56 (37)	2.6 (1.4–4.6)	0.001	37 (24)	4.4 (2.4–8.0)	<10^−4^
• cotrimoxazole	9 (6)	5 (3)	2.0 (0.6–6.6)	0.3	8 (5)	1.1 (0.4–3.1)	0.8
• fluoroquinolones	17 (11)	10 (7)	1.9 (0.8–4.4)	0.2	5 (3)	3.4 (1.2–9.2)	0.02
• penicillins	36 (24)	24 (16)	1.6 (0.9–2.8)	0.1	19 (13)	2.1 (1.1–3.8)	0.02
• extended spectrum cephalosporins	18 (12)	17 (11)	1.1 (0.5–2.1)	0.9	11 (7)	1.9 (0.8–4.4)	0.2
• aminoglycosides	13 (9)	12 (8)	1.1 (0.5–2.6)	0.8	2 (1)	6.5 (1.5–28.8)	0.01
• carbapenems	9 (6)	3 (2)	3.0 (0.8–11.9)	0.1	0	-	-
• ≥5 days	48 (32)	25 (16)	3.3 (1.6–6.7)	9×10^−4^	29 (13)	3.6 (1.9–7.1)	1×10^−4^
Specimen and infection data							
- specimen sampled after 48 h of hospitalisation.	86 (57)	92 (61)	0.8 (0.5–1.4)	0.4	63 (41)	1.9 (1.2–3.1)	0.008
- specimen sampled after >10 days of hospitalisation.	56 (37)	51 (33)	1.2 (0.7–1.9)	0.5	31 (20)	2.8 (1.5–5.1)	0.001
- urine sample	97 (64)	104 (68)	0.8 (0.5–1.3)	0.4	81 (53)	1.6 (1.0–2.6)	0.07
- urinary tract infection	100 (66)	104 (68)	0.9 (0.5–1.4)	0.6	-	-	-

ICU: intensive care unit, LTCF: long-term care facility.

In multivariate analysis (Table 4), variables independently associated with a CTX-M-producing *E. coli* isolate were country of birth outside of Europe (OR = 2.4; 95% = 1.3–4.5), recurrent UTI or chronic skin infections (OR = 2.9; 95%CI = 1.3–6.9), previous hospitalisation (OR = 2.0; 95%CI = 1.1–3.6), antibiotic treatment and ICU hospitalisation between admission and inclusion (OR = 2.0; 95%CI = 1.0–3.8, and OR = 2.3; 95%CI = 1.1–5.0, respectively).

**Table 4:** Multivariate analysis of factors associated with a CTX-M-producing *E. coli* clinical isolate.

Independent variable	Odds ratio (95% CI)	P value
**Comparison with controls C1**		
*Demographic data*		
Country of birth outside of Europe	2.4 (1.3–4.5)	0.004
*Medical history*		
Recurrent urinary tract or chronic skin infections	2.9 (1.3–6.9)	0.01
Hospitalised in the preceding 6 months	2.0 (1.1–3.6)	0.01
*Current hospitalisation*		
Having been or being in ICU during the current hospitalisation.	2.3 (1.1–5.0)	0.03
Antibiotic receipt between admission and inclusion.	2.0 (1.0–3.8)	0.04
**Comparison with controls C2**		
*Demographic data*		
Country of birth outside of Europe	3.1 (1.4–6.9)	0.005
Female gender	2.5 (1.2–5.2)	0.02
*Lifestyle*		
Functionally dependent before hospitalisation.	7.0 (2.1–23.5)	0.002
*Medical history*		
Recurrent urinary tract or chronic skin infections	8.7 (1.9–39.7)	0.005
Urine drainage in the preceding 6 months	4.4 (1.6–11.5)	0.003
*Current hospitalisation*		
At least one invasive device between admission and inclusion.	4.2 (1.6–10.8)	0.003
Antibiotic receipt between admission and inclusion.	3.3 (1.5–7.2)	0.003

ICU: intensive care unit.

### Second case-control study

When cases were compared to C2, univariate analysis showed that living in a country outside of Europe, contact with pets or livestock, cotrimoxazole or extended-spectrum cephalosporin treatments, and an antibiotic treatment ≥5 days were no longer associated with isolation of CTX-M-producing *E. coli* (Tables 1 and 2). However, 16 additional factors were identified (Tables 1 and 2): age ≥80 years (OR = 2.1; 95%CI = 1.1–4.0), female gender (OR = 1.7; 95%CI = 1.1–2.8), collective housing (OR = 10.4; 95%CI = 2.4–44.8), functional dependence before hospitalisation (OR = 5.1; 95%CI = 2.3–11.6), previous hospitalisation in another country than France (OR = 8.0; 95%CI = 1.0–64.0), previous fluoroquinolone treatment (OR = 3.7; 95%CI = 1.0–13.1), and at least one comorbidity (OR = 2.1; 95%CI = 1.4–3.4). The nine remaining factors were linked to the current hospitalisation (Table 3). Of interest, living alone (Table 1) was inversely associated with isolation of CTX-M-producing *E. coli* (OR = 0.4; 95%CI = 0.2–0.8)

In the multivariate analysis comparing cases and C2 (Table 4), four factors not identified in the first multivariate analysis comparing cases to C1 were found independently associated with isolation of CTX-M-producing *E. coli*: female gender (OR = 2.5; 95%CI = 1.2–5.2), functional dependence before hospitalisation (OR = 7.0; 95%CI = 2.1–23.5), previous urinary drainage (OR = 4.4; 95%CI = 1.6–11.5), and at least one invasive device between admission and inclusion (OR = 4.2; 95%CI = 1.6–10.8).

### Subpopulation analysis of cases with an imported CTX-M-producing *E. coli* isolate

Among the 66 cases with a CTX-M-producing *E. coli* clinical sample detected within the first 48 h of hospitalisation (*i.e.* imported), only 33 had a C1 for whom the non-ESBL-producing *E. coli* isolate was detected within the same time frame, whereas all had a C2. In univariate analysis, the 33 cases were more likely than C1 (Table 5) to live in collective housing (OR = 5.0; 95%CI = 1.1–22.8); to have a previous hospitalisation (OR = 4.9; 95%CI = 1.4–17.3), and at least one invasive device in the last six months (OR = 7.0; 95%CI = 1.6–30.8). All these variables, except for previous hospitalisation, were also significantly associated with an imported CTX-M-producing *E. coli* isolate when the 66 cases were compared to C2 (Table 6). Moreover, the cases-C2 comparison showed that functional dependence before hospitalisation (OR = 4.0; 95%CI = 1.1–14.2), and antibiotic treatment in the last month (OR+2.5; 95%CI = 1.1–5.7) were also associated factors (Table 6). Finally, consumption of raw meat was inversely associated (OR = 0.4; 95%CI = 0.2–0.9) with an imported CTX-M-producing *E. coli* isolate (Table 6).

**Table 5:** Univariate analysis of factors associated with a CTX-M producing *E. coli* clinical isolates within the first 48 h of hospitalisation in subpopulations of cases (patients with a CTX-M producing *E. coli* isolate) and controls (C1: patients with a non-ESBL producing *E. coli* isolate).

Factor*	No.(%) of Cases n = 33	No. (%) of C1 n = 33	Odds ratio (95% CI)	P value
*Demographic data*				
Country of birth outside of Europe	8 (24)	6 (18)	1.5 (0.4–5.3)	0.5
Living in a country outside of Europe	1 (3)	0	-	-
*Lifestyle*				
Collective housing	16 (48)	8 (24)	5.0 (1.1–22.8)	0.03
Consumption of raw meat	9 (27)	15 (45)	0.4 (0.1–1.3)	0.1
Pets or livestock	4 (12)	4 (12)	1.0 (0.2–4.0)	1.0
*Medical history*				
In the preceding 6 months				
- hospitalised	20 (60)	8 (24)	4.9 (1.4–17.3)	0.01
- hospitalised ≥10 days	10 (30)	4 (12)	3.0 (0.8–11.1)	0.09
- hospitalised <10 days	10 (30)	4 (12)	4.0 (0.8–18.8)	0.08
- at least one invasive device	23 (70)	11 (33)	7.0 (1.6–30.8)	0.01
• urine drainage	12 (37)	4 (12)	5.0 (1.1–22.8)	0.04
• intravascular devices	22 (69)	9 (28)	5.0 (1.5–17.3)	0.01
Antibiotic receipt in the month preceding hospitalisation.	11 (33)	4 (12)	2.7 (0.9–8.6)	0.08
- cotrimoxazole	3 (9)	0	-	-
- extended spectrum cephalosporins	5 (15)	1 (13)	5.0 (0.6–42.8)	0.2
- ≥5 days	4 (12)	2 (6)	2.0 (0.4–11)	0.4
Recurrent urinary tract or chronic skin infections	5 (15)	3 (9)	2.0 (0.4–10.9)	0.4

Variables presented here comprised those found with a p value (p<0.1) in the comparison between the 33 cases and their controls C1 and those found significant (p≤0.05) in the univariate analysis performed for all cases in comparison with their controls C1.

**Table 6:** Univariate analysis of factors associated with a CTX-M producing *E. coli* clinical isolate within the first 48 h of hospitalisation in subpopulations of cases (patients with a CTX-M producing *E. coli* isolate) and controls (C2 : patients with negative clinical samples).

Factor*	No. (%) of Cases n = 66	No. (%) of C2 n = 66	Odds ratio (95% CI)	P value
*Demographic data*				
Age ≥80 years	19 (29)	15 (23)	1.6 (0.6–4.0)	0.3
Female	44 (67)	37 (56)	1.6 (0.8–3.2)	0.2
Country of birth outside of Europe	18 (27)	11 (17)	1.7 (0.8–3.7)	0.2
*Lifestyle*				
Collective housing	10 (15)	1 (2)	10.0 (1.3–78.1)	0.03
Functionally dependent before hosp.	14 (21)	5 (8)	4.0 (1.1–14.2)	0.03
Live alone	16 (24)	26 (36)	0.6 (0.2–1.2)	0.1
Patients not working	46 (70)	36 (55)	2.1 (1.0–4.7)	0.06
Retired patients	38 (58)	27 (41)	2.2 (1.0–4.9)	0.05
Consumption of raw meat	17 (26)	31 (47)	0.4 (0.2–0.9)	0.01
Pets or livestock	8 (12)	17 (26)	0.4 (0.2–1.1)	0.07
*Medical history*				
In the preceding 6 months				
- hospitalised	42 (64)	33 (50)	1.9 (0.9–4.1)	0.1
- hospitalised ≥10 days	25 (38)	18 (27)	1.8 (0.8–4.0)	0.2
- hospitalised in another country than France	5 (8)	0	-	-
- at least one invasive device	47 (71)	35 (53)	2.3 (1.1–5.1)	0.03
• urine drainage	23 (36)	10 (16)	3.0 (1.2–7.6)	0.02
• intravascular device	45 (69)	31 (48)	2.2 (1.0–4.6)	0.04
Antibiotic receipt in the month preceding hosp.	24 (36)	12 (18)	2.5 (1.1–5.7)	0.03
At least one co-morbidity	33 (50)	23 (35)	1.8 (0.9–3.5)	0.1
- recurrent urinary tract or chronic skin infections	10 (15)	1 (2)	-	-

Variables presented here comprised those found with a p value (p<0.1) in the comparison between the 66 cases and their controls C2 and those found significant (p≤0.05) in the univariate analysis performed for all cases in comparison with their controls C2.

In multivariate analysis, factors independently associated with an imported CTX-M-producing *E. coli* isolate were to have at least one invasive device in the last six months (OR = 7.0; 95%CI = 1.6–30.8), when comparing cases to C1, and collective housing (OR = 15.2; 95%CI = 1.8–130.7) and intravascular devices in the last six months (OR = 2.9; 95%CI = 1.2–6.9), when comparing cases to C2.

## Discussion

CTX-M-producing *E. coli* are spreading worldwide, and it is suggested that transmission occurs mainly in the community [2]. Therefore, besides classical factors linked to MDR bacteria carriage, the present study focused on variables reflecting patient's lifestyle and history before hospitalisation that may expose to CTX-M-producing *E. coli* in the community. Thus, we showed that country of birth was significantly associated with isolation of *E. coli* producing CTX-M. Moreover, being born outside of Europe was identified in the two case-control studies. Ninety six percent of foreign-born cases originated from Africa and Asia, two regions well known for immigration to France. The rather elevated median age of foreign-born cases (64 years) suggests that these immigrants have been living in France for several years. In addition, the proportion of recent travel among cases was quite low (11%) and travel abroad was not significantly associated with isolation of CTX-M-producing *E. coli* [9]. Therefore, the hypothesis that recent travels of foreign-born cases to their country of birth with a high prevalence of ESBL-producing *Enterobacteriaceae* does not seem to hold [10]–[13]. However, foreign-born cases are more likely than French-born cases to be in contact with recent immigrants or relatives living in countries with high prevalence of ESBL, this may increase the exposition of the former to MDR bacteria cross-transmission [14]. Specific studies should be conducted to address this hypothesis.

The second case-control study (cases *vs* C2) demonstrated for the first time that functional dependence before hospitalisation was also associated with isolation of CTX-M- producing *E. coli*. This suggests that the need for living assistance at home may promote cross-transmission by close contact between cases and relatives or professionals and subsequently increases the risk for bacterial transmission.

Of interest, chronic infections were found as associated with isolation of CTX-M-producing *E. coli*. Chronic infections might lead to repeated use of antibiotics. Such repeated regimens may enhance the persistence and the predominance of resistant bacteria such as CTX-M-producing *E. coli* in the digestive flora and promote thereafter extra-intestinal infections. This hypothesis is reinforced by the fact that the two case-control studies linked antibiotic use to a higher risk of isolation of CTX-M-producing *E. coli.* However, in multivariate analysis, the link involved antibiotics received after hospital admission and not those received within the month preceding admission. This point has never been identified obviously because differentiating between these two time-periods of antibiotic exposure was seldom done in previous studies. Of note, the use of antibiotic during both periods was identified as a risk factor for CTX-M-producing *E. coli* detection in univariate analysis. The lack of relationship between antibiotic use during the month preceding admission and CTX-M-producing *E. coli* through the multivariate analysis suggests that, either, the more recent the antibiotic use (i.e. administered during hospitalisation), the stronger the association would be, or other factors independently associated with CTX-M-producing *E. coli* are more prominent than antibiotic use before hospitalisation.

Most other factors identified in the present study are clearly related to the healthcare system, suggesting that hospital setting is a reservoir of *E. coli* producing CTX-M. On the opposite, collective housing, which was associated with an imported CTX-M producing *E. coli* isolate, suggests that a reservoir of CTX-M-producing *E. coli* exists outside of the hospital setting. From this point of view, a high proportion of residents of Irish nursing homes has been found to carry ESBL-producing *E. coli* [15]. Therefore, collective housing as well as the other risk-factors of isolation of CTX-M-producing *E. coli* should be part of the standard medical interview of patients suspected of infection.

Finally, the present study failed to identify numerous factors previously associated with a clinical sample positive for ESBL-producing *E. coli* such as travel abroad [9], co-morbidities [9], [16], [17], transfer from another hospital [18], or previous use of oxyimino β-lactams [19]–[21].

In addition, our population-based study did not identify diet or food habits as a factor associated with CTX-M-producing *E. coli*. On the contrary, raw meat consumption was shown to be inversely associated with CTX-M-producing *E. coli* in univariate analysis, which seems to be in contradiction with recent data showing raw chicken meat to be contaminated by CTX-M-producing *E. coli* [22], [23]. However, consumption of contaminated raw chicken meat as a source of CTX-M-producing *E. coli* in humans has never been demonstrated. Raw meat consumption in our studied population is likely to be a surrogate for French-born status because the populations at risk in our study are foreign-born persons that are used to eating well-cooked meat.

To our knowledge, this study is the first prospective, multicentre case-control-control study on factors associated with CTX-M-producing *E. coli* in any type of clinical specimens obtained either within or after the first 48 h of hospitalisation. Indeed, most previous studies were retrospective or cohort studies [9], [16], [20], [24]–[27]. In addition, previous case-control studies compared mostly cases to a single control group comprising patients with a clinical sample positive for a non-ESBL-producing micro-organism [17], [21], [28]. The use of two control-groups, which is a strength of our study, has two main advantages [29]. First, identifying the same risk-factors in two case-control studies reinforces the strength of the association. Second, the ability to identify various risk-factors is increased by the use of different control populations. Following this principle, Rodriguez-Bano *et al.* performed two case-control studies on the same topic, and used two comparison groups [19], [30]. However, they focused, as many other studies, on only one type of infection, especially bacteraemia [16], [19], [20], [28], [30], or UTI [21], [26], while we did not select for the type of infection, which is a second strength of our study. Finally, we investigated both community- and hospital-onset infections on the contrary to previous studies, which is the third strength of our study. All these differences may explain the differences observed between our study and the others regarding factors associated with isolation of CTX-M-producing *E. coli*.

Our study has some limitations. We did not search for asymptomatic faecal carriage in all control-patients. Therefore, some of these patients may have been misclassified preventing from identifying some risk-factors. Collective housing has been assessed as a single factor preventing from identifying specific populations within this group such as nursing homes or retirement homes. Finally, antibiotic exposure prior to hospitalisation was assessed for only one month. Antibiotic exposure could have been assessed for a longer period of time (6 to 12 months). However, the accuracy of information provided on antibiotic treatment is likely to decrease with the time span and is subject to memory bias.

In conclusion, this prospective case-control-control study identified three types of factors associated with CTX-M-producing *E. coli*: those related to the patient medical history, those related to care provided during hospitalisation, and those associated with patient's origin and lifestyle. These new non-healthcare-related factors, together with those previously identified, such as travel abroad, warrant further studies in order to get more insight into the epidemiology of CTX-M-producing *E coli*, notably in the community, that is a new real public health concern in both developed and developing countries.

## Methods

### Ethical approval

The study was approved by the Ethics Committee of the Groupe Hospitalier Universitaire Nord (Institutional review board N°IRB00006477).

### Study design and participants

The study was carried out from November 2008 to June 2009 in ten hospitals of AP-HP (7 554 beds), including short- (n = 5) and long- (n = 2) term care facilities as well as paediatric hospitals (n = 3). Factors associated with a clinical sample positive for CTX-M-producing *E. coli* in patients hospitalised for at least 24 h, was studied by using a case-control-control design. We followed the methodological principles recommended for case-control studies that analyse risk factors for antibiotic resistance, *i.e.* controls derived from the same source population as cases and selected during the same time periods [29]. Moreover, two different control groups were selected in order to get a better representation of the total base population.

Patients prospectively identified by the microbiological laboratory of each participating hospital with a clinical sample yielding ESBL-producing *E. coli* were eligible for the study. To be pre-included (pre-case), eligible patients had to be still hospitalised, able to answer a standardised questionnaire, and to have control-patients. For each pre-case, two controls were selected on the laboratory register of the same hospital. The first control (C1) was the first inpatient with a clinical sample positive for a non-ESBL-producing *E. coli* the same day or within the three days following the pre-case detection. The second control (C2) was the first inpatient with specimen(s) negative for bacterial growth since admission until three days after the pre-case detection. Pre-cases were included (cases), as well as their controls, when the ESBL enzyme was characterised as CTX-M. An isolate was deemed imported in the hospital when it was detected within the first 48 h of hospitalisation. Otherwise, it was considered as hospital-acquired. Written informed consent was obtained from all adult cases and controls and from parents for child cases and controls.

### Variables

One hundred and fourteen variables were prospectively collected by two investigators in the ten hospitals from inpatients, their family, medical team, and the bacteriological and medical files for all cases and controls.

#### Demographic data

Standard demographic data, including country of birth and place of residence were collected.

#### Patient's lifestyle

Living arrangement was divided into three categories: individual housing with one or two household members, individual housing with more than two, and collective housing (college dormitories, homeless schelters, shelters for immigrants, homes for young workers, retirement homes and nursing homes). Patients receiving assistance for daily living before the current hospitalisation were considered functionally dependent. Occupation, unemployment, or retirement was clarified. Data on diet were grouped into four categories according to the type of food. Meal outside home was recorded. Sport practice, spa use during the last six months, contact with animals, travel abroad during the last six months were recorded. If appropriate, chronic infections and potential exposure to MDR *E. coli* (occupation related to health or in a healthcare setting and hospitalisation in the last six months) of household members were collected.

#### Medical history

The following variables were collected: hospitalisation, length of hospital stay, and invasive devices during the preceding six months; prosthesis during the last year; surgery, home care, and antimicrobial treatment during the last month. In addition, co-morbidities (recurrent UTI, chronic skin infections, obstructive bronchial pulmonary disease, cancer, diabetes or dialysis) were assessed.

#### Current hospitalisation

Dates of admission in the hospital and in the ward in which cases and controls were included, type of admission (direct or transfer), date of discharge, and in-hospital death were recorded. The Mc Cabe score was used as a proxy for underlying illness [31]. A patient was considered immunocompromised if he was under immunosuppressive drugs *i.e.* chemotherapy, radiotherapy, or corticosteroids (≥30 days or >5 mg/kg for 5 days); he had haematological disease, metastatic cancer or HIV-related CD4<500 mm3. Presence of invasive devices within the last week, and antibacterial treatment between hospital admission and inclusion in the study were documented. Date of sampling and type of clinical specimens positive for CTX-M-producing *E. coli* (case) or non-ESBL-producing *E. coli* (C1) or culture-negative samples (C2) were collected.

### Microbiological analysis

*E. coli* isolates were locally identified by using the API 20E system (bioMérieux, Marcy l'Etoile, France). ESBL production was detected by the double disk synergy test routinely applied in each laboratory as recommended by the French Antibiogram Committee (http://www.sfm.asso.fr/nouv/general.php?pa=2). [32], [33] *E. coli* strains screened as ESBL producers were sent to three laboratories of the ten participating hospitals and sub-cultured on chromogenic media (bioMérieux). ESBL-encoding *bla* genes were searched for by PCR as previously described [34]. Then, the amplified fragments were sent to Institut Pasteur in Paris to be sequenced by using primers specific for *bla*_CTX-M_, *bla*_TEM_ and *bla*_SHV_ genes, as previously described [34].

### Study size

The study size was derived from the total number of patients with ESBL-producing *E. coli* available through an active surveillance programme implemented in each hospital of AP-HP since more than 15 years [35]. In addition, characterization of the type of enzyme produced by ESBL-producing *E. coli* was performed in 2005, and thus allowing to evaluate the proportion of CTX-M-producing *E. coli* among all ESBL-producing *E. coli*. Therefore, approximately 260 patients with an ESBL-producing *E .coli* isolate were expected during a 6-month study period, including 180 with *E. coli* producing CTX-M. With regard to a risk factor present in 10% of controls, this number of included cases and controls will allow to detect an odds ratio of 3 with a power of 90% and a type 1 error of 5%. For a risk factor present in 20% of controls, an odds ratio of 2.4 is expected.

### Statistical analysis

For the main objective, comparisons were analysed between cases and C1 and then between cases and C2. Variables associated with cases were analysed using conditional logistic regression on the pairs of 152 cases and their controls. Odds ratios and 95% confidence intervals were first estimated in univariate analysis. Variables with a p-value <0.1 were introduced into the multivariate analysis and were selected thereafter by using a backward selection method. The same method was applied to cases that had a CTX-M-producing *E. coli* isolated from a specimen sampled within the first 48 h after hospitalisation, *i. e.* imported cases, and their controls which also had a specimen sampled during the same period. All statistical analyses were performed with SAS software, version 9.1 (SAS Institute, Cary, North Carolina). P-values were assessed at the 0.05 level.
